# Physical Health of Young, Australian Women: A Comparison of Two National Cohorts Surveyed 17 Years Apart

**DOI:** 10.1371/journal.pone.0142088

**Published:** 2015-11-03

**Authors:** Ingrid J Rowlands, Annette J Dobson, Gita D Mishra

**Affiliations:** Centre for Longitudinal and Life Course Research, The University of Queensland, School of Public Health, Herston, QLD, 4006, Australia; Bambino Gesù Children's Hospital, ITALY

## Abstract

**Introduction:**

Very little is known about the extent of physical health issues among young women in early adulthood and whether this is changing over time.

**Methods:**

We used data from two national samples of young women aged 18–23 years, surveyed 17 years apart, who participated in the Australian Longitudinal Study on Women’s Health. We used multinomial logistic regression to compare the women’s physical health (i.e., self-rated health, common symptoms and conditions) and identify whether sociodemographic factors, health behaviours and stress explained any physical health differences between the samples.

**Results:**

Women aged 18–23 years in 2013 (N = 17,069) were more likely to report poor self-rated health and physical symptoms (particularly urogenital and bowel symptoms) than women aged 18–23 years in 1996 (N = 14,247). Stress accounted for a large proportion of the physical health differences between the cohorts, particularly for allergies, headaches, self-rated health, severe tiredness, skin problems, severe period pain and hypertension.

**Conclusions:**

Women’s health appears to be changing, with young women born in more recent decades reporting greater physical symptom levels. Changing socio-cultural and economic conditions may place pressure on young adults, negatively affecting their health and wellbeing. Assessing the extent to which social structures and health care policies are offering adequate support to young women may offer avenues for promoting positive health and wellbeing.

## Introduction

Although people in developed countries are living longer, there is evidence to suggest that their health and wellbeing is becoming worse, not better [1, 2]. Much of the research focuses on adult health, however, studies from several countries suggest that the decline in health and wellbeing is most apparent among adolescents and young adults. Adolescent girls and young women appear to be at greater risk of poor health and wellbeing than boys and young men [3–5], with studies showing that physical symptoms (e.g. headaches, tiredness) and emotional distress are increasing [6–8]. The reasons for the apparent health “decline” are complex but are often attributed to individual health (e.g., obesity) and health behaviours (e.g., smoking) and socioecominic factors (e.g., unemployment, education). Sociocultural factors are less often considered [2, 9], but Western cultural values that emphasise wealth, materialism and individualism may place pressure on young adults, negatively affecting their health and wellbeing [2, 10].

Although some stress is normal, and often expected, for young people as they transition into adulthood [11, 12], increasing social pressures may negatively impact on adolescent and adult health [13]. Adolescent worries about school achievement and family conflict have been associated with increasing rates of psychological distress and stress [5, 14]. Similarly, adults’ concerns about work and family are associated with physical symptoms [15]. Current understandings and perceptions of health and illness are also shaped by social context. For example, Western culture has been criticised for “medicalising” and “pathologising” “everyday problems” [16], particularly among women, leading to an overdiagnosis of physical and mental health conditions [17]. At the same time, there will be young women who have significant physical and mental health issues [17] and early intervention is a priority. However, there is very little evidence regarding the extent of physical health issues among young women in early adulthood and whether this is in fact changing over time.

In this paper we compare the physical health (i.e., self-rated health, common symptoms and conditions) of two national samples of young, Australian women (aged 18–23 years) who were surveyed 17 years apart. We aim to identify whether sociodemographic factors, health behaviours and stress may explain any physical health differences between the cohorts.

## Methods

The Australian Longitudinal Study on Women’s Health (ALSWH) is a national study focusing on the biological, psychological, social and economic factors relevant to women’s health [18]. The original three cohorts of Australian women, who were aged 18–23 years, 45–50 years, and 70–75 years when the project began in 1996, are sent self-report surveys on a triennial basis to explore their health and wellbeing [18]. The 40,000 participants were randomly selected using the national health insurance database (Medicare), which includes all permanent residents of Australia. Women in rural and remote area were oversampled to ensure that their health and health service needs could be adequately assessed. Comparisons with Australian census data show that the three cohorts of women are broadly representative of the Australian population in these age groups [19].

In 2012–13, ALSWH recruited a new cohort of young women born 1989–95 and aged 18–23 when they were first surveyed. Participants were recruited through conventional (e.g., magazine advertising) and online social media avenues (e.g., Facebook) and completed a web-based survey similar to previous ALSWH surveys sent to the young women (full details reported elsewhere) [20, 21]. Women were eligible if they lived in Australia, had a valid Medicare number and if they consented to linking survey data with administrative health data. Further details of the survey methodology are available from the study website [22].

### Ethics statement

Participants were asked to provide formal, written consent to their data being linked to administrative datasets and to provide contact details and their Medicare number to facilitate data linkage. Data were sent to the Australian Department of Human Services to verify participants’ Medicare numbers and personal details. Informed participant consent was implied by survey initiation. All study methods, including the consent procedures, were approved by the Human Research Ethics Committees of the University of Newcastle, the University of Queensland, the Australian Department of Human Services and the Australian Department of Health.

### Participants

This paper uses data collected from two samples of young women, surveyed 16–17 years apart, when both groups were aged 18–23 years. The analysis includes 14,247 young women born in 1973–78 who responded to a baseline survey in 1996 and 17,069 women born in 1989–95 who responded to a baseline survey in 2012–13.

### Measures

#### Outcomes

In both surveys, women were asked to rate their general health (excellent, very good, good, fair or poor) using an item from the 36-item Short Form health survey (SF-36) [23], and to report if they had ever been diagnosed with a chronic health condition (e.g. asthma, hypertension, low iron), classified as “yes” vs. “no”. Women were also asked about the frequency of 15 symptoms (never, rarely, sometimes, often) in the last 12 months: Somatic symptoms (e.g. headaches, severe tiredness, back pain), urogenital and menstrual symptoms (e.g., urine that burns or stings, leaking urine, heavy periods, severe period pain), digestive and intestinal symptoms (e.g., constipation, hemorrhoids, other bowel problems).

#### Sociodemographics

Sociodemographic information was also collected from both cohorts and included age (in years); area of residence based on an index of distance to the nearest urban centre (major cities, inner regional, outer regional, remote/very remote) [24]; highest level of education (less than year 12, year 12 or equivalent, certificate/diploma, university degree); current relationship status (never married, never married but in a relationship, married/engaged, separated/divorced/widowed) and parity (yes, no).

#### Health behaviours

Both cohorts of women were asked about smoking (current smokers, ex-smokers or never smokers) and alcohol consumption (categorised according to the National Health and Medical Research Council Australian Alcohol Guidelines) [25]. Women reported their height (cm) and body weight (kg), which was used to calculate body mass index (BMI) and classified according to the World Health Organization’s guidelines: underweight (<18.5 kg/m^2^), normal weight (18.5 to <25 kg/m^2^), overweight (25 to <30 kg/m^2^) and obese (≥30 kg/m^2^) [26].

#### Stress

Women’s level of perceived stress over the last 12 months in specific areas of their life including study, relationships and their own health was measured using the Perceived Stress Questionnaire for Young Women [27]. The questionnaire is used to derive an overall mean stress score, ranging in from 0 (no stress) to 4 (extreme stress). The Perceived Stress Questionnaire is correlated with other psychological and physical health measures and has moderate internal reliability with Cronbach’s alpha being 0.75 [28, 29].

### Statistical Analysis

Multinomial logistic regression was used to compare the physical health of the 1989–95 cohort (18–23 years in 2013) to 1973–78 cohort (18–23 years in 1996). Potential confounding variables were added to the models in blocks to assess their association with physical health outcomes: Sociodemographic variables (age, marital status, education, area of residence, parity), followed by health behaviours (BMI, alcohol consumption, smoking) and then stress. Age (in single years), area of residence and education were included to account for design and response differences between the samples. An interaction term between education and cohort was also included in the models because the association between cohort and physical health differed by education.

Data analysis was conducted using SAS software, version 9.4 (TS1M0) of the SAS System for Windows [30].

## Results

The 1989–95 cohort of women were similar to the 1973–78 cohort regarding sociodemographic characteristics: Most were not married, residing in a major city and had no children. There was slight over-representation of better-educated and non-smoking women in the 1989–95 cohort, and a greater proportion of women were obese and reported higher levels of stress (see Table 1).

**Table 1:** Characteristics of two cohorts of young Australian women who were aged 18–23 years when first surveyed in 1996 and in 2013.

	18–23 years in 1996 N = 14,247	18–23 years in 2013 N = 17,069		
	N(%)	N(%)	χ^2^	*P*-value
**Age**				
18–19	2308 (17.1)	5585 (32.8)	969.52	<.0001
20–21	5735 (42.5)	5733 (33.6)		
22–23	5464 (40.5)	5730 (33.6)		
**Marital Status**				
Single	10850 (76.5)	12134 (71.9)	95.85	<.0001
Married/defacto	3193 (22.5)	4616 (27.3)		
Separated/divorced/other	134 (0.9)	134 (0.8)		
**Education**				
<Year 12	2427 (17.1)	1271 (7.5)	1586.67	<.0001
Year 12	7600 (53.6)	7341 (43.5)		
Certificate/Diploma	2563 (18.1)	4428 (26.2)		
University	1576 (11.1)	3844 (22.8)		
**Area of residence**				
Major city	7375 (51.8)	12849 (75.5)	1950.25	<.0001
Inner regional	4307 (30.3)	2831 (16.6)		
Outer regional	2090 (14.7)	1151 (6.8)		
Remote	465 (3.3)	183 (1.1)		
**Parity**				
No	12777 (90.4)	15898 (94.2)	165.01	<.0001
Yes	1245 (8.7)	975 (5.8)		
**Body mass Index (BMI)**				
Underweight (<18.5)	1245 (8.7)	1332 (7.8)	2300.96	<.0001
Healthy weight (18.5–24.9)	8361 (58.7)	9923 (58.1)		
Overweight (25–29.9)	1875 (13.2)	3231 (18.9)		
Obese (≥30)	772 (5.4)	2298 (13.5)		
Missing	1994 (14.0)	285 (1.7)		
**Alcohol consumption**				
Low risk drinker	7197 (50.5)	9370 (54.9)	405.01	<.0001
Non-drinker/ Rarely drinks	6109 (42.9)	6364 (37.3)		
Risky/High risk drinker	782 (5.5)	626 (3.7)		
Missing	159 (1.1)	709 (4.2)		
**Smoking**				
Never	7123 (50.0)	10629 (62.3)	1095.44	<.0001
Ex-smoker	2085 (14.6)	3081 (18.1)		
Current	4421 (31.0)	3191 (18.7)		
Missing	618 (4.3)	168 (0.98)		
**Stress**				
*M (SD)*	0.89 (0.57)	1.27 (0.63)	-55.27^a^	<.0001

^a^two sample *t*-test

Compared to the 1973–78 cohort, the 1989–95 cohort appeared to be in poorer physical health. They were more likely to report their self-rated general health as “fair or poor”; and to report allergies, headaches, severe tiredness, back pain and skin problems “often” (see Table 2). Tables 3 and 4 show that women in the 1989–95 cohort also had higher odds of urogenital and menstrual symptoms—particularly leaking urine, vaginal discharge and irregular periods—and constipation, hemorrhoids, other bowel problems. They also had higher odds of low iron but lower odds of hypertension and cancer (see Table 5). With the exception of premenstrual tension and leaking urine, adjustment for sociodemographic characteristics and health behaviours did not make substantive changes to the results.

**Table 2:** Comparisons of somatic symptoms between two cohorts of young women aged 18 to 23 years in 1996 and in 2013. Odds ratios for participants in 2013 compared to participants in 1996.

			18–23 years in 2013			
	18–23 years in 1996	18–23 years in 2013		Sociodemographics	Health behaviours	Stress
	N(%)	N(%)	UnadjustedOR (95% CI)	Adjusted^a^OR (95% CI)	Adjusted^b^OR (95% CI)	Adjusted^c^OR (95% CI)
**Self-rated general health**						
Excellent/Very Good	7242 (51.1)	7178 (42.5)	1	1	1	1
Good	5208 (36.8)	6866 (40.6)	1.33 (1.27–1.40)	1.34 (1.25–1.44)	1.38 (1.28–1.49)	1.12 (1.03–1.21)
Fair/Poor	1716 (12.1)	2859 (16.9)	1.68 (1.57–1.80)	1.71 (1.55–1.90)	1.73 (1.53–1.93)	1.13 (1.01–1.27)
**Allergies**						
Never	5831 (41.2)	6146 (36.4)	1	1	1	1
Rarely	2675 (18.9)	3139 (18.6)	1.11 (1.05–1.19)	1.02 (0.93–1.12)	1.02 (0.93–1.13)	0.93 (0.84–1.02)
Sometimes	3396 (24.0)	4283 (25.3)	1.20 (1.13–1.27)	1.17 (1.07–1.27)	1.19 (1.09–1.30)	1.07 (0.98–1.17)
Often	2249 (15.9)	3333 (19.7)	1.41 (1.32–1.50)	1.28 (1.16–1.40)	1.22 (1.11–1.35)	0.99 (0.90–1.10)
**Headaches/migraines**						
Never	1454 (10.2)	1317 (7.8)	1	1	1	1
Rarely	4551 (32.1)	4861 (28.8)	1.18 (1.08–1.28)	1.08 (0.96–1.23)	1.08 (0.95–1.23)	1.00 (0.88–1.14)
Sometimes	5530 (39.0)	6915 (40.9)	1.38 (1.27–1.50)	1.23 (1.09–1.39)	1.23 (1.08–1.39)	0.98 (0.86–1.11)
Often	2655 (18.7)	3809 (22.5)	1.58 (1.45–1.73)	1.40 (1.23–1.60)	1.40 (1.22–1.60)	0.93 (0.81–1.07)
**Severe tiredness**						
Never	3033 (21.4)	1773 (10.5)	1	1	1	1
Rarely	3982 (28.1)	3712 (22.0)	1.60 (1.48–1.72)	1.46 (1.31–1.63)	1.51 (1.35–1.69)	1.28 (1.14–1.43)
Sometimes	4565 (32.2)	6445 (38.1)	2.42 (2.25–2.59)	2.30 (2.07–2.55)	2.47 (2.22–2.75)	1.75 (1.56–1.95)
Often	2601 (18.3)	4966 (29.4)	3.27 (3.03–3.52)	3.01 (2.69–3.36)	3.31 (2.95–3.71)	1.85 (1.64–2.10)
**Back pain**						
Never	4678 (33.0)	2982 (17.6)	1	1	1	1
Rarely	4040 (28.5)	4688 (27.7)	1.82 (1.71–1.94)	1.79 (1.63–1.96)	1.85 (1.68–2.03)	1.64 (1.49–1.80)
Sometimes	3752 (26.4)	5674 (33.6)	2.37 (2.23–2.52)	2.46 (2.25–2.69)	2.62 (2.38–2.88)	2.13 (1.94–2.35)
Often	1724 (12.1)	3556 (21.0)	3.24 (3.01–3.48)	3.21 (2.87–3.58)	3.58 (3.19–4.02)	2.50 (2.22–2.82)
**Skin problems**						
Never	6344 (44.7)	4721 (27.9)	1	1	1	1
Rarely	3324 (23.4)	4631 (27.4)	1.87 (1.77–1.98)	1.64 (1.50–1.79)	1.64 (1.50–1.79)	1.46 (1.33–1.60)
Sometimes	2666 (18.8)	4503 (26.6)	2.27 (2.14–2.41)	2.13 (1.94–2.33)	2.11 (1.92–2.32)	1.78 (1.62–1.95)
Often	1858 (13.1)	3048 (18.0)	2.20 (2.06–2.36)	1.88 (1.70–2.08)	1.88 (1.69–2.08)	1.45 (1.30–1.61)

^a^Adjusted for age, education, area of residence, marital status, and parity

^b^Adjusted for age, education, area of residence, marital status, parity, BMI, alcohol and smoking

^c^Adjusted for age, education, area of residence, marital status, parity, BMI, alcohol, smoking and stress

**Table 3:** Comparisons of urogenital and menstrual symptoms between two cohorts of young women aged 18 to 23 years in 1996 and in 2013. Odds ratios for participants in 2013 compared to participants in 1996.

			18–23 years in 2013			
	18–23 years in 1996	18–23 years in 2013		Sociodemographics	Health behaviours	Stress
	N(%)	N(%)	UnadjustedOR (95% CI)	Adjusted^a^OR (95% CI)	Adjusted^b^OR (95% CI)	Adjusted^c^OR (95% CI)
**Urine that burns or stings**						
Never	10186 (71.8)	10566 (62.5)	1	1	1	1
Rarely	2676 (18.9)	4105 (24.3)	1.48 (1.40–1.56)	1.59 (1.47–1.73)	1.67 (1.53–1.82)	1.43 (1.31–1.56)
Sometimes	1094 (7.7)	1897 (11.2)	1.67 (1.54–1.81)	1.84 (1.64–2.07)	1.99 (1.76–2.24)	1.53 (1.35–1.73)
Often	234 (1.6)	327 (1.9)	1.35 (1.14–1.60)	1.35 (1.05–1.74)	1.50 (1.15–1.95)	1.05 (0.80–1.37)
**Leaking urine**						
Never	12312 (86.7)	11958 (70.8)	1	1	1	1
Rarely	1222 (8.6)	2957 (17.5)	2.49 (2.32–2.68)	2.73 (2.46–3.04)	2.78 (2.49–3.11)	2.41 (2.16–2.70)
Sometimes	522 (3.7)	1601 (9.5)	3.16 (2.85–3.50)	3.34 (2.86–3.89)	3.45 (2.94–4.05)	2.77 (2.36–3.26)
Often	144 (1.0)	383 (2.3)	2.74 (2.26–3.32)	3.64 (2.66–4.98)	3.60 (2.61–4.98)	2.52 (1.82–3.50)
**Vaginal discharge**						
Never	7981 (56.3)	4301 (25.4)	1	1	1	1
Rarely	3555 (25.1)	5443 (32.2)	2.84 (2.69–3.01)	3.07 (2.82–3.34)	3.30 (3.03–3.60)	2.94 (2.69–3.21)
Sometimes	2048 (14.4)	5329 (31.5)	4.83 (4.53–5.14)	5.17 (4.71–5.67)	5.76 (5.22–6.34)	4.69 (4.25–5.18)
Often	604 (4.3)	1830 (10.8)	5.62 (5.09–6.21)	5.59 (4.83–6.46)	6.32 (5.44–7.35)	4.75 (4.07–5.53)
**Premenstrual tension**						
Never	4632 (32.7)	4606 (27.3)	1	1	1	1
Rarely	3178 (22.4)	4114 (24.3)	1.30 (1.22–1.39)	1.10 (1.00–1.20)	1.09 (0.99–1.20)	0.96 (0.87–1.06)
Sometimes	4035 (28.5)	5428 (32.1)	1.35 (1.28–1.43)	1.22 (1.12–1.33)	1.20 (1.10–1.31)	0.93 (0.85–1.02)
Often	2325 (16.4)	2750 (16.3)	1.19 (1.11–1.27)	0.99 (0.89–1.10)	1.00 (0.90–1.12)	0.66 (0.59–0.74)
**Heavy periods**						
Never	6767 (47.7)	5638 (33.4)	1	1	1	1
Rarely	3500 (24.7)	4535 (26.8)	1.56 (1.47–1.65)	1.55 (1.42–1.68)	1.59 (1.46–1.74)	1.40 (1.28–1.53)
Sometimes	2588 (18.3)	4062 (24.0)	1.88 (1.77–2.00)	1.76 (1.61–1.93)	1.85 (1.69–2.03)	1.53 (1.39–1.68)
Often	1325 (9.3)	2668 (15.8)	2.42 (2.24–2.60)	2.33 (2.08–2.60)	2.41 (2.15–2.71)	1.79 (1.59–2.01)
**Severe period pain**						
Never	4861 (34.3)	4247 (25.1)	1	1	1	1
Rarely	3655 (25.8)	4373 (25.9)	1.37 (1.29–1.45)	1.27 (1.16–1.39)	1.31 (1.19–1.43)	1.15 (1.05–1.27)
Sometimes	3297 (23.3)	4485 (26.5)	1.56 (1.47–1.66)	1.42 (1.30–1.56)	1.48 (1.34–1.62)	1.19 (1.08–1.31)
Often	2367 (16.7)	3794 (22.5)	1.84 (1.72–1.96)	1.56 (1.42–1.72)	1.65 (1.50–1.83)	1.19 (1.07–1.33)
**Irregular periods**						
Never	8277 (58.4)	6383 (37.8)	1	1	1	1
Rarely	2461 (17.4)	3605 (21.3)	1.90 (1.79–2.02)	1.80 (1.65–1.97)	1.86 (1.69–2.04)	1.66 (1.51–1.82)
Sometimes	1829 (12.9)	3503 (20.7)	2.48 (2.33–2.65)	2.32 (2.11–2.56)	2.42 (2.19–2.67)	2.08 (1.88–2.30)
Often	1612 (11.4)	3410 (20.2)	2.74 (2.56–2.93)	2.51 (2.27–2.77)	2.64 (2.39–2.93)	2.09 (1.88–2.33)

^a^Adjusted for age, education, area of residence, marital status, and parity

^b^Adjusted for age, education, area of residence, marital status, parity, BMI, alcohol and smoking

^c^Adjusted for age, education, area of residence, marital status, parity, BMI, alcohol, smoking and stress

**Table 4:** Comparison of digestive and intestinal symptoms between two cohorts of young women aged 18 to 23 years in 1996 and in 2013. Odds ratios for participants in 2013 compared to participants in 1996.

			18–23 years in 2013			
	18–23 years in 1996	18–23 years in 2013		Sociodemographics	Health behaviours	Stress
	N(%)	N(%)	UnadjustedOR (95% CI)	Adjusted^a^OR (95% CI)	Adjusted^b^OR (95% CI)	Adjusted^c^OR (95% CI)
**Constipation**						
Never	8627 (60.8)	6704 (39.7)	1	1	1	1
Rarely	3548 (25.0)	5707 (33.8)	2.07 (1.96–2.18)	2.04 (1.89–2.21)	2.11 (1.99–2.24)	1.84 (1.69–1.99)
Sometimes	1501 (10.6)	3485 (20.6)	2.99 (2.79–3.20)	3.23 (2.91–3.57)	3.29 (3.03–3.56)	2.77 (2.48–3.09)
Often	507 (3.6)	1003 (5.9)	2.55 (2.28–2.85)	2.67 (2.25–3.17)	2.84 (2.50–3.23)	2.06 (1.72–2.46)
**Haemorrhoids**						
Never	13175 (92.9)	14824 (87.7)	1	1	1	1
Rarely	564 (4.0)	1090 (6.4)	1.72 (1.55–1.91)	1.95 (1.66–2.29)	2.02 (1.72–2.39)	1.82 (1.54–2.16)
Sometimes	329 (2.3)	692 (4.1)	1.87 (1.64–2.14)	2.45 (1.96–3.06)	2.50 (1.99–3.13)	2.15 (1.71–2.71)
Often	121 (0.9)	294 (1.7)	2.16 (1.75–2.67)	3.04 (2.09–4.42)	2.98 (2.03–4.37)	2.38 (1.61–3.50)
**Other bowel problems**						
Never	12372 (87.2)	11258 (66.6)	1	1	1	1
Rarely	972 (6.9)	2872 (17.0)	3.25 (3.01–3.51)	3.14 (2.81–3.51)	3.26 (2.91–3.66)	2.86 (2.54–3.21)
Sometimes	549 (3.9)	1876 (11.1)	3.76 (3.40–4.14)	3.89 (3.37–4.50)	4.08 (3.51–4.73)	3.28 (2.82–3.82)
Often	292 (2.1)	895 (5.3)	3.37 (2.94–3.85)	3.23 (2.64–3.95)	3.43 (2.79–4.23)	2.64 (2.13–3.26)

^a^Adjusted for age, education, area of residence, marital status, and parity

^b^Adjusted for age, education, area of residence, marital status, parity, BMI, alcohol and smoking

^c^Adjusted for age, education, area of residence, marital status, parity, BMI, alcohol, smoking and stress

**Table 5:** Comparison of chronic conditions between two cohorts of young women aged 18 to 23 years in 1996 and in 2013. Odds ratios for participants in 2013 compared to participants in 1996.

			18–23 years in 2013			
	18–23 years in 1996	18–23 years in 2013		Sociodemographics	Health behaviours	Stress
	N(%)		Unadjusted OR (95% CI)	Adjusted^a^ OR (95% CI)	Adjusted^b^ OR (95% CI)	Adjusted^c^ OR (95% CI)
**Hypertension**						
No	13453 (94.9)	16832 (98.6)	1	1	1	1
Yes	722 (5.1)	237 (1.4)	0.27 (0.23–0.31)	0.27 (0.20–0.34)	0.24 (0.18–0.31)	0.19 (0.15–0.25)
**Low iron**						
No	10614 (75.0)	11743 (68.8)	1	1	1	1
Yes	3537 (25.0)	5326 (31.2)	1.38 (1.31–1.45)	1.44 (1.34–1.56)	1.57 (1.45–1.69)	1.34 (1.23–1.45)
**Asthma**						
No	10613 (74.9)	12724 (74.5)	1	1	1	1
Yes	3561 (25.1)	4345 (25.5)	1.03 (0.98–1.09)	0.99 (0.92–1.07)	0.98 (0.91–1.07)	0.88 (0.82–0.96)
**Cancer**						
No	13869 (98.4)	16956 (99.3)	1	1	1	1
Yes	228 (1.6)	113 (0.7)	0.41 (0.33–0.51)	0.45 (0.32–0.65)	0.49 (0.34–0.71)	0.41 (0.29–0.60)

^a^Adjusted for age, education, area of residence, marital status, and parity

^b^Adjusted for age, education, area of residence, marital status, parity, BMI, alcohol and smoking

^c^Adjusted for age, education, area of residence, marital status, parity, BMI, alcohol, smoking and stress

Adding stress to the models did account for a large proportion of the physical health differences between the cohorts. The estimates for allergies and headaches were not significant when adjusting for stress. In addition, stress attenuated the differences between the cohorts for self-rated health, severe tiredness, skin problems, severe period pain and hypertension, and to a lesser extent, asthma and other bowel problems. The odds of having back pain, urine that burns or stings, vaginal discharge, heavy periods and irregular periods “often” were also lower for women in the 1989–95 cohort after adjustment for stress. However, adjustment for stress did not alter the estimates for cancer, low iron and vaginal discharge.

## Discussion

The results of this study, comparing two national cohorts of young Australian women when they were both aged 18–23 years, suggest that young women’s health is declining. Women born in 1989–95 were more likely to reported poor self-rated health and physical symptoms (particularly urogenital and bowel symptoms) than women born in 1973–78. Of the young women who were born in 1989–95, less than half rated their health as “excellent” or “good”, which is notably lower than what has been reported for 15–24 year olds in the 2011–12 Australian National Health Survey (63% rate health as “excellent” or “good”) [31]. The decline in health may occur early, with a meta-analysis of studies examining the health of children and adolescents reporting an “excess” of physical health symptoms among girls compared to boys, largely for headache, abdominal pain, tiredness, migraine and self-rated health [4].

Poor health among young women may increase the risk of chronic illness in late adulthood and, thus, identifying potential “causes” is necessary for prevention and intervention. While sociodemographic characteristics and health behaviors are associated with physical health outcomes [32], health behaviors explained relatively little of the difference between the health of the cohorts of women in this study. The transition from adolescence to adulthood is marked by engagement in “risky” behaviors (e.g., smoking, alcohol and illicit drug use) [11, 33], and with the exception of the decline in smoking, many of these behaviors have not changed over time among our cohorts of women. Instead, women’s level of perceived stress in various areas of their life attenuated the odds of poorer physical health in the 1989–95 cohort.

Researchers suggest that changing socio-cultural and economic conditions are part of the complex web of factors influencing young adults’ health [2, 10]. Young women are more stressed than men [34], which may be the product of “conflicts and ambiguities” for women that are not socially imposed on young men [12]. The decline in young women’s health is not unique to Australia, with several other international studies reporting poor health and wellbeing among young women. Many of these studies are from Sweden [5, 7, 8, 10], which is highly regarded for its health care system and policies [35]. This evidence perhaps points to societal rather than health care deficits explaining the decline in young women’s health.

Socio-cultural and medical understandings of health and illness and individual factors (e.g., age, health experiences and expectations) contribute to a person’s perception of their health and also influence with whom (e.g. peers, family, general population) people use to compare their health status [36–38]. In addition, the rapidly changing medical landscape in Australia, including increased screening, diagnosis and availability of medication may make people more aware of, and focused on, their health. Together, these factors may increase social expectations about what constitutes ‘good’ health and this may explain some of the apparent self-reported decline in health in the 1989–95 cohort.

Although women born in 1989–95 did not have higher odds of chronic health conditions, they were more likely to report high symptom levels. Because many of the common symptoms examined in this study are also those that are frequently experienced by people in times of heightened stress, disentangling cause and effect can be difficult [13]. However, viewing stress as part of, rather than separate from, poor physical health perhaps offers a way to understand these findings. Similarly, broad definitions and measures of health that do not that assume “health” only in the absence of major disease, will be more effective at capturing the extent of health issues relevant to young people [2, 39].

In this study we were able to compare the health of two, national cohorts of women within the same age group, using similar measures, which offers an initial step towards understanding if, and why, young Australian women’s health is changing. While it is possible that recent social, economic and health service changes in Australian society (i.e., period effects) explain the differences in physical symptoms between the two samples, we are unable to disentangle period effects from cohort effects in this study due to the absence of longitudinal data for the 1989–95 cohort [40]. In addition, some measures between our surveys were not compatible (e.g. physical activity, psychological distress; diabetes), and others were not available (e.g., social support) [41], and these variables may assist further in explaining the differences between the cohorts. Although the survey administration also differed between the cohorts (paper versus Internet), there is evidence that the reliability and validity of measures of health and wellbeing do not differ between these survey administration modes [42, 43]. Women’s health appears to be changing, with young women born in more recent decades reporting greater physical symptom levels. Some of the “excess” may be explained by higher levels of perceived stress among our recent cohort of young women, and this may have long-term consequences on women’s mental health as well as major life transitions [44]. Measuring societal changes in epidemiologic surveys is challenging but feasible with cross-cohort comparisons such as the present study. Assessing the extent to which social structures and health care policies are offering adequate support to young women may offer avenues for promoting positive health and wellbeing.

## References

[1] Eckersley RM. The health and well-being of young Australians: present patterns and future challenges. International journal of adolescent medicine and health. 2007 Jul-Sep;19(3):217–27. . 17937137 10.1515/ijamh.2007.19.3.217

[2] Eckersley R. A new narrative of young people's health and well-being. Journal of Youth Studies. 2011 2011/08/01;14(5):627–38.

[3] Laftman SB, Modin B, Ostberg V, Hoven H, Plenty S. Effort-reward imbalance in the school setting: Associations with somatic pain and self-rated health. Scandinavian journal of public health. 2014 12 10 . 25504584 10.1177/1403494814561818

[4] MacLean A, Sweeting H, Egan M, Der G, Adamson J, Hunt K. How robust is the evidence of an emerging or increasing female excess in physical morbidity between childhood and adolescence? Results of a systematic literature review and meta-analyses. Social science & medicine. 2013 2;78:96–112. . Pubmed Central PMCID: 3566587. 23273876 10.1016/j.socscimed.2012.11.039 PMC3566587

[5] Östberg V, Almquist Y, Folkesson L, Låftman S, Modin B, Lindfors P. The Complexity of Stress in Mid-Adolescent Girls and Boys. Child Ind Res. 2014 2014/04/19:1–21. English.

[6] Bor W, Dean AJ, Najman J, Hayatbakhsh R. Are child and adolescent mental health problems increasing in the 21st century? A systematic review. The Australian and New Zealand journal of psychiatry. 2014 5 14;48(7):606–16. 10.1177/0004867414533834 24829198

[7] Friberg P, Hagquist C, Osika W. Self-perceived psychosomatic health in Swedish children, adolescents and young adults: an internet-based survey over time. BMJ open. 2012;2(4). 22855621 10.1136/bmjopen-2011-000681 PMC4400616

[8] Wiklund M, Malmgren-Olsson EB, Ohman A, Bergstrom E, Fjellman-Wiklund A. Subjective health complaints in older adolescents are related to perceived stress, anxiety and gender—a cross-sectional school study in Northern Sweden. BMC public health. 2012;12:993 Pubmed Central PMCID: 3533931. 10.1186/1471-2458-12-993 23158724 PMC3533931

[9] Marks DF. Health Psychology in Context. Journal of Health Psychology. 1996 1 1, 1996;1(1):7–21. 10.1177/135910539600100102 22011517

[10] Wiklund M, Bengs C, Malmgren-Olsson EB, Ohman A. Young women facing multiple and intersecting stressors of modernity, gender orders and youth. Social science & medicine. 2010 11;71(9):1567–75. 20846769 10.1016/j.socscimed.2010.08.004

[11] Arnett JJ. Adolescent storm and stress, reconsidered. The American psychologist. 1999 5;54(5):317–26. . 10354802 10.1037//0003-066x.54.5.317

[12] Bell S, Lee C. Transitions in emerging adulthood and stress among young Australian women. International journal of behavioral medicine. 2008;15(4):280–8. 10.1080/10705500802365482 19005927

[13] Carver CS, Vargas S. Stress, coping, and health. The Oxford Handbook of Health Psychology. 2011:162.

[14] Sweeting H, West P, Young R, Der G. Can we explain increases in young people's psychological distress over time? Social science & medicine. 2010 11;71(10):1819–30. . Pubmed Central PMCID: 2981856. 20870334 10.1016/j.socscimed.2010.08.012 PMC2981856

[15] Dorner TE, Stronegger WJ, Rebhandl E, Rieder A, Freidl W. The relationship between various psychosocial factors and physical symptoms reported during primary-care health examinations. Wiener klinische Wochenschrift. 2010 2;122(3–4):103–9. Epub 2010/03/10. eng. 10.1007/s00508-010-1312-6 20213377

[16] Double D. The limits of psychiatry2002 2002-04-13 07:00:00. 900–4 p.

[17] Ussher JM. Are We Medicalizing Women’s Misery? A Critical Review of Women’s Higher Rates of Reported Depression. Feminism & Psychology. 2010 2 1, 2010;20(1):9–35.

[18] Lee C, Dobson AJ, Brown WJ, Bryson L, Byles J, Warner-Smith P, et al Cohort Profile: the Australian Longitudinal Study on Women's Health. International journal of epidemiology. 2005 10;34(5):987–91. . 15894591 10.1093/ije/dyi098

[19] Brown WJ, Dobson AJ, Bryson L, Byles JE. Women's Health Australia: on the progress of the main cohort studies. Journal of women's health & gender-based medicine. 1999 6;8(5):681–8. . 10839654 10.1089/jwh.1.1999.8.681

[20] Loxton D, Powers J, Anderson A, Townsend N, Harris M, Tukerman R, et al. Recruiting young women to a longitudinal health survey in the 21st century: findings from the Australian Longitudinal Study on Women’s Health 1989–95 cohort. Unpublished manuscript.

[21] Mishra GD, Hockey R, Powers J, Loxton D, Tooth L, Rowlands I, et al Recruitment via the Internet and social networking sites results in a representative sample of young women: the 1989–95 cohort of the Australian Longitudinal Study on Women’s Health. Journal of Medical Internet Research 2014;15;16(12):e279. 10.2196/jmir.3788 PMC4275491 25514159

[22] Australian Longitudinal Study on Women's Health (ALSWH). [October 2014]. Available from: http://alswh.org.au/for-participants/1989-95-cohort

[23] Ware JE Jr., Sherbourne CD. The MOS 36-item short-form health survey (SF-36). I. Conceptual framework and item selection. Medical care. 1992 6;30(6):473–83. . 1593914

[24] Australian Institute of Health and Welfare (AIHW). Rural, regional and remote health: a guide to remoteness classifications. Canberra: AIHW, 2004.

[25] National Health and Medical Research Council. Australian guidelines to reduce health risks from drinking alcohol Canberra: Commonwealth of Australia; 2009.

[26] WHO. Obesity: preventing and managing the global epidemic. Geneva: WHO, 2000. 11234459

[27] Bell S, Lee C. Development of the Perceived Stress Questionnaire for Young Women. Psychology, Health & Medicine. 2002 2002/05/01;7(2):189–201.

[28] Bell S, Lee C. Perceived stress revisited: The Women's Health Australia project Young cohort. Psychology, Health & Medicine. 2003 2003/08/01;8(3):343–53.

[29] Bell S, Lee C. Does timing and sequencing of transitions to adulthood make a difference? Stress, smoking, and physical activity among young Australian women. International journal of behavioral medicine. 2006;13(3):265–74. . 17078778 10.1207/s15327558ijbm1303_11

[30] Inc. SI. SAS/STAT 13.2 User’s Guide. Cary, NC: SAS Institute Inc.; 2014.

[31] Australian Bureau of Statistics (ABS). 4364.0.55.003—Australian Health Survey: Updated Results, 2011–2012 Canberra, Australia: ABS; 2013. Available from: http://www.abs.gov.au/ausstats/abs@.nsf/Lookup/4364.0.55.003Chapter1002011-2012.

[32] Lantz PM, House JS, Lepkowski JM, Williams DR, Mero RP, Chen J. Socioeconomic factors, health behaviors, and mortality: results from a nationally representative prospective study of US adults. Jama. 1998 6 3;279(21):1703–8. . 9624022 10.1001/jama.279.21.1703

[33] Eaton DK, Kann L, Kinchen S, Shanklin S, Flint KH, Hawkins J, et al Youth risk behavior surveillance-United States, 2011. Morbidity and mortality weekly report Surveillance summaries (Washington, DC: 2002). 2012;61(4):1–162. 22673000

[34] Cohen S, Janicki-Deverts D. Who's Stressed? Distributions of Psychological Stress in the United States in Probability Samples from 1983, 2006, and 20091. Journal of Applied Social Psychology. 2012;42(6):1320–34.

[35] Nolte E, McKee M. Measuring the health of nations: analysis of mortality amenable to health care. Bmj. 2003 11 15;327(7424):1129 . Pubmed Central PMCID: 261807. 14615335 10.1136/bmj.327.7424.1129 PMC261807

[36] Jylha M. What is self-rated health and why does it predict mortality? Towards a unified conceptual model. Social science & medicine. 2009 8;69(3):307–16. . 19520474 10.1016/j.socscimed.2009.05.013

[37] Wolff LS, Subramanian SV, Acevedo-Garcia D, Weber D, Kawachi I. Compared to whom? Subjective social status, self-rated health, and referent group sensitivity in a diverse US sample. Social science & medicine. 2010 6;70(12):2019–28. . Pubmed Central PMCID: 3571719. 20381225 10.1016/j.socscimed.2010.02.033 PMC3571719

[38] Kaplan G, Baron-Epel O. What lies behind the subjective evaluation of health status? Social science & medicine. 2003 4;56(8):1669–76. 12639584 10.1016/s0277-9536(02)00179-x

[39] Thacker SB, Stroup DF, Carande-Kulis V, Marks JS, Roy K, Gerberding JL. Measuring the public's health. Public health reports. 2006 Jan-Feb;121(1):14–22. . Pubmed Central PMCID: 1497799. 16416694 10.1177/003335490612100107 PMC1497799

[40] Reither EN, Hauser RM, Yang Y. Do birth cohorts matter? Age-period-cohort analyses of the obesity epidemic in the United States. Social science & medicine. 2009 11;69(10):1439–48. Pubmed Central PMCID: 2782961. 19773107 10.1016/j.socscimed.2009.08.040 PMC2782961

[41] Thoits PA. Mechanisms linking social ties and support to physical and mental health. Journal of health and social behavior. 2011 6;52(2):145–61. 10.1177/0022146510395592 21673143

[42] Touvier M, Mejean C, Kesse-Guyot E, Pollet C, Malon A, Castetbon K, et al Comparison between web-based and paper versions of a self-administered anthropometric questionnaire. European journal of epidemiology. 2010 5;25(5):287–96. 10.1007/s10654-010-9433-9 20191377

[43] Basnov M, Kongsved SM, Bech P, Hjollund NH. Reliability of short form-36 in an Internet- and a pen-and-paper version. Informatics for health & social care. 2009 1;34(1):53–8. . 19306199 10.1080/17538150902779527

[44] Lee C, Gramotnev H. Life transitions and mental health in a national cohort of young Australian women. Developmental psychology. 2007 7;43(4):877–88. . 17605521 10.1037/0012-1649.43.4.877

